# Relevance of matrix metalloproteases in non-small cell lung cancer diagnosis

**DOI:** 10.1186/s12885-017-3842-z

**Published:** 2017-12-05

**Authors:** Sonia Blanco-Prieto, Leticia Barcia-Castro, María Páez de la Cadena, Francisco Javier Rodríguez-Berrocal, Lorena Vázquez-Iglesias, María Isabel Botana-Rial, Alberto Fernández-Villar, Loretta De Chiara

**Affiliations:** 1 Department of Biochemistry, Genetics and Immunology, Universidade de Vigo.Vigo, As Lagoas-Marcosende s/n, 36310 Vigo, Spain; 2 Department of Pneumology of Hospital Álvaro Cunqueiro EOXI Vigo, Carretera Clara Campoamor, 341, 36212 Vigo, Spain

**Keywords:** Matrix metalloproteases, Non-small cell lung cancer, Diagnosis, Serum biomarkers

## Abstract

**Background:**

The need for novel biomarkers that could aid in non-small cell lung cancer (NSCLC) detection, together with the relevance of Matrix Metalloproteases (MMPs) -1, -2, -7, -9 and -10 in lung tumorigenesis, prompted us to assess the diagnostic usefulness of these MMPs and the Tissue Inhibitor of Metalloproteinase (TIMP) -1 in NSCLC patients.

**Methods:**

Markers were evaluated in an initial study cohort (19 NSCLC cases and 19 healthy controls). Those that better performed were analyzed in a larger sample including patients with benign lung diseases. Serum MMPs and TIMP-1 were determined by multiplexed immunoassays. Logistic regression was employed for multivariate analysis of biomarker combinations.

**Results:**

MMPs and TIMP-1 were elevated in the serum of NSCLC patients compared to healthy controls. MMP-1, -7 and -9 performed at best and were further evaluated in the sample including benign pathologies, corroborating the superiority of MMP-9 in NSCLC discrimination, also at early-stage NSCLC. The optimal diagnostic value was obtained with the model including MMP-9, gender, age and smoking history, that demonstrated an AUC of 0.787, 85.54% sensitivity and 64.89% specificity.

**Conclusion:**

Our results suggest that MMP-9 is a potential biomarker for NSCLC diagnosis and its combined measurement with other biomarkers could improve NSCLC detection.

**Electronic supplementary material:**

The online version of this article (10.1186/s12885-017-3842-z) contains supplementary material, which is available to authorized users.

## Background

Non-small cell lung cancer (NSCLC) accounts for 75–80% of the newly diagnosed lung cancers and includes the main histological subtypes adenocarcinoma (ADC), squamous cell carcinoma (SCC) and large cell carcinoma (LCC) [1]. NSCLC 5-year survival rates around 13% [2] make essential an improvement of prognosis, which can be achieved with the detection of cancer at early stages. Consequently, there is an imperative need of non-invasive tests, preferably blood-based biomarkers that could be used as tools for the early detection of lung cancer [3, 4].

Matrix metalloproteases (MMPs) constitute a large family of structurally related, zinc- and calcium-dependent enzymes capable of degrading almost all of the extracellular matrix (ECM) proteins. These endopeptidases have been widely associated with the development of various diseases, including cancer [5]. The over-expression of MMPs induced by both tumor cells and surrounding stroma is not limited to matrix degradation, favoring invasion and metastasis (reviewed in [6, 7]). Through the activation of non-matrix substrates such as growth factors, cytokines and other membrane proteins, MMPs are also involved in initial stages of tumor development mediating signaling pathways related to cell migration, differentiation, proliferation, apoptosis, angiogenesis and inflammatory reactions [8].

Numerous studies have demonstrated that MMPs are specifically implicated in lung-tumorigenesis driven processes, contributing to the formation of a complex microenvironment promoting malignant transformation in lung tissue (reviewed in [9]). MMP-1 has proved to be a tumor growth promoting and pro-angiogenic factor in the lungs of MMP-1-deficient mice [10]. MMP-2 holds a role in lung cancer angiogenesis mediated by vascular endothelial growth factor expression [11] and also in the invasive behavior of tumor cells, as for MMP-7 [12]. A common role in metastasis has been attributed to MMP-9, triggered by MMP-9 induction in the premetastatic lung by other distant primary tumors [13]. Regarding MMP-10, over-expression in cancer-initiating or cancer stem cells is key on their maintenance and tumorigenic potential, allowing for lung tumor-initiating activity and metastatic spread [14].

The functions of MMPs are controlled by the four Tissue Inhibitors of Metalloproteinases, TIMP-1 − 4 [15]. The local balance between MMPs and TIMPs is critical to avoid the conditions of uncontrolled ECM turnover, inflammation, and deregulated cell growth and migration, which would result in disease [16].

The involvement of the aforementioned MMPs and their inhibitors in lung carcinogenesis makes this group of molecules attractive as potential markers for NSCLC. Our objective was to determine the serum levels of the selected MMPs (MMP-1, -2, -7, -9 and -10) and TIMP-1 in NSCLC patients and healthy and benign controls to investigate their capability for NSCLC discrimination.

## Methods

### Study population

Patients with respiratory symptoms were prospectively enrolled at the Department of Pneumology of Hospital Álvaro Cunqueiro EOXI Vigo (Spain) between June 2007 and June 2011. Diagnosis and classification of lung cancer patients were based on the clinical guidelines of the American College of Chest Physicians [17]. Patients with non-NSCLC histology, relapse or progression of a previously diagnosed cancer, or administration of radiotherapy/chemotherapy were excluded from the study.

Initial evaluation of MMPs and TIMP-1 was conducted in a reduced cohort of 19 NSCLC cases and 19 healthy controls, whose demographics are presented in Additional file 1. Markers exhibiting greater discriminative capability were determined in a larger sample set including 193 individuals: 83 NSCLC cases (48.2% ADC, 31.3% SCC and 16.9% LCC) and 110 controls (75 individuals with benign lung pathology and 35 healthy patients). Characteristics of NSCLC patients and controls are summarized in Table 1. Smoking status was defined ‘Yes’ for current smokers and ex-smokers, and ‘No’ for never-smokers.

**Table 1:** Patient demographics

	Cases (*n* = 83)	Controls (*n* = 110)
Gender^a^		
Male	68 (81.9%)	62 (56.4%)
Female	15 (18.1%)	48 (43.6%)
Age^b^		
Median	69	61
Range	42–88	24–88
Smoking status^c^		
Yes	74 (89.2%)	62 (66%)
No	9 (10.8%)	32 (33%)
Diagnosis		
Healthy		35
Benign Pathology		75
RI		53 (70.7%)
ILD		17 (22.6%)
Nodule		4 (5.3%)
ICC		1 (1.3%)
NSCLC Histology		
ADC	40 (48.2%)	
SCC	26 (31.3%)	
LCC	14 (16.9%)	
BAC	2 (2.4%)	
ND	1 (1.2%)	
NSCLC Stage		
I	18 (21.7%)	
II	6 (7.2%)	
III	22 (26.5%)	
IV	37 (44.6%)	

*RI* Respiratory Infection, *ILD* Interstitial Lung Disease, *ICC* Congestive Heart Failure, *NSCLC* Non-Small Cell Lung Cancer, *ADC* Adenocarcinoma, *SCC* Squamous Cell Carcinoma, *LCC* Large Cell Carcinoma, *BAC* Bronchioloalveolar Carcinoma, *ND* Not Differentiated Carcinoma

^a^Gender distribution between cancer and controls statistically significant: *P* < 0.001 (Fisher test)

^b^Statistically significant differences in age between cancer and controls: *P* = 0.001 (Mann-Whitney U test)

^c^Smoking status distribution between cancer and controls statistically different: *P* < 0.001 (Fisher test)

Blood samples from all patients and controls were collected at their first visit to the Department of Pneumology and serum was separated and stored at −20 °C until analysis. The study was conducted in compliance with the clinical-ethical practices of the Spanish Government and the Helsinki Declaration, and Galician Ethical Committee for Clinical Research approved the protocol. Written informed consent from each patient was obtained.

### Measurement of serum MMPs and TIMP-1 concentration

Serum MMPs and TIMP-1 determination was carried out by means of multiplexed immunoassays with Luminex xMAP technology (*EMD Millipore*, Missouri, USA). Measurements of MMP-1, MMP-2, MMP-7, MMP-9 and MMP-10 were conducted with the commercially available Human MMP Panel 2 Magnetic Bead kit (HMMP2MAG, *EMD Millipore*) according to the manufacturer protocol, while TIMP-1 was part of the Human TIMP Panel 1 Magnetic Bead kit (HTMP1MAG, *EMD Millipore*).

Fluorescence readings were collected on a Luminex platform (Luminex 200™), and calculation of results was performed using the BioPlex Manager ™ software (Bio-Rad, Hercules, CA), with protein concentrations calculated using a 5-parametric curve fitting. Both standard and serum samples were assayed in duplicate to reduce variation.

### Statistical methods

Descriptive statistics were obtained for continuous (median and range) and categorical variables (frequencies). Differences in serum biomarker concentrations were assessed using the non-parametric Mann-Whitney U test. The Fisher exact test was applied to determine the association between qualitative variables. To analyze the diagnostic accuracy of the biomarkers for NSCLC diagnosis, Receiver Operating Characteristic (ROC) curves were calculated, providing the area under the ROC curve (AUC). *P*-values ≤0.05 were considered statistically significant.

Univariate logistic regression was performed to evaluate the individual capability of biomarkers and demographic variables of predicting lung malignancy. Logistic regression models, using log10-tranformed marker concentrations to reduce skewness, were employed for multivariate analysis of biomarker combinations. Models were constructed with all possible combinations of selected markers to determine the optimal marker set. Age and gender were included in the regression models to adjust for confounding. Predicted probabilities of malignancy for each individual, generated by the logistic function, were used to calculate the diagnostic performance of the assayed marker combinations, providing the AUC and the sensitivity and specificity based on the Youden index. Statistical analyses were carried out with the statistical software SPSS 15.0 (SPSS Inc., Chicago, IL); sensitivity and specificity were calculated using the MedCalc software.

## Results

### Analysis of serum MMPs and TIMP-1 in NSCLC patients and healthy controls: Initial study set

Members of the MMP family MMP-1, -2, -7, -9 and -10 and the MMP-9 inhibitor TIMP-1 were first assayed in the initial study set to select the molecules with best performance.

All MMPs and TIMP-1 exhibited elevated levels in cancer patients (Table 2). However, concentrations were only significantly higher for MMP-1 (*P* = 0.015), MMP-9 (*P* < 0.001) and MMP-9/TIMP1 ratio (*P* = 0.002), despite MMP-7 showed notably higher levels in NSCLC. ROC curves proved the superiority of MMP-9 for discriminating NSCLC cases with an AUC of 0.852, followed by MMP-9/TIMP1 (AUC 0.781), MMP-1 (AUC 0.729) and MMP-7 with a modest discriminatory capacity (AUC 0.658). Correction of MMP-9 levels by its inhibitor levels failed to improve the diagnostic performance of the matrix metalloproteinase member, therefore TIMP1 was not further evaluated.

**Table 2:** Serum Levels of MMPs and TIMP-1 in Non-Small Cell Lung Cancer and Healthy Controls in the Initial Study Set

Marker^a^	Median	Range	*P* ^b^	AUC (95% CI)
MMP-1 (pg/mL)				
Healthy	4504.00	1186.61–16,751.12	0.015	0.729 (0.560–0.897)
NSCLC	8739.00	2517.57–20,715.00		
MMP-2 (pg/mL)				
Healthy	84,644.00	76,006.00–117,129.00	0.584	0.446 (0.251–0.641)
NSCLC	85,643.00	60,249.00–144,375.00		
MMP-7 (pg/mL)				
Healthy	16,964.00	11,409.14–27,067.99	0.120	0.658 (0.473–0.843)
NSCLC	20,742.11	7256.00–50,354.00		
MMP-9 (ng/mL)				
Healthy	136.14	63.13–492.91	<0.001	0.852 (0.725–0.979)
NSCLC	302.92	131.16–824.08		
MMP-10 (pg/mL)				
Healthy	350.00	132.00–993.00	0.533	0.564 (0.355–0.774)
NSCLC	400.00	209.00–730.00		
TIMP-1 (ng/mL)				
Healthy	130.45	92.40–238.90	0.191	0.625 (0.443–0.806)
NSCLC	151.65	110.05–241.00		
MMP-9/TIMP1				
Healthy	1.09	0.51–5-05	0.002	0.781 (0.628–0.934)
NSCLC	2.42	0.73–4.80		

*NSCLC* Non-Small Cell Lung Cancer

^a^Sample size: Healthy *N* = 19, NSCLC *N* = 19

^b^Mann-Whitney U test for the comparison between cancer and healthy group

### Analysis of serum MMP-1, -7 and -9 in NSCLC and control patients

After identifying MMP-1, -7 and -9 as the MMPs with major contribution to NSCLC detection, they were further evaluated in a larger set consisting of 83 NSCLC cases, 35 healthy controls and 75 patients with benign lung pathology.

The relationship between demographic variables and marker concentrations were established, as presented in Table 3. Analysis showed significant differences in serum MMP-9 regarding gender, with levels being higher in males (*P* = 0.010). In relation to age, MMP-7 levels were found to be significantly higher in older individuals (*P* < 0.001). Smoking history did not significantly influence levels of any of the markers, although notable higher MMP-9 concentrations occurred in smokers in relation to subjects who have never smoked.

**Table 3:** Relationship Between Serum Levels of MMP-1, −7 and −9 and Demographic Parameters

Marker	Gender^a^			Age^a^			Smoking History^a, c^		
	Male (*n* = 130)	Female (*n* = 63^c^)	*P* ^b^	≤64 years (*n* = 97)	>64 years (*n* = 96)	*P* ^b^	Yes (*n* = 136)	No (*n* = 41)	*P* ^b^
MMP-1 (pg/mL)	6061.80	7572.03	0.799	6146.60	6384.94	0.682	5872.18	7683.08	0.700
	1207.70–41,668.33	935.32–24,851.96		1081.81–32,677.57	935.32–41,668.33		1271.24–41,668.33	935.32–24, 851.96	
MMP-7 (pg/mL)	24,238.56	23,035.24	0.777	20,810.85	27,700.03	<0.001	24,411.99	22,974.55	0.349
	5026.14–79,977.27	5383.18–72,191.16		5026.14–72,191.16	5383.18–79,977.27		5026.14–79,977.27	5383.18–59,232.82	
MMP-9 (ng/mL)	273.18	201.75	0.010	228.04	260.42	0.477	265.73	201.75	0.060
	21.06–3611.59	11.07–3300.50		11.07–3611.59	52.79–1883.70		21.06–3611.59	11.07–3300.50	

^a^Median and range values provided

^b^Mann-Whitney U test for the comparison between gender, age and smoking groups

Statistically significant higher serum levels were confirmed for MMP-7 (*P* = 0.014) and MMP-9 (*P* < 0.001) when comparing lung cancer with non-cancer patients (both healthy and benign). Increased concentration of MMP-1 in benign controls, compared to healthy individuals, accounts for the lack of significance. In addition, when NSCLC and benign controls were compared, only MMP-9 resulted significantly altered (*P* < 0.001). These findings, including the median values for markers in the different diagnostic groups, are provided in Table 4.

**Table 4:** Serum Levels of MMP-1, −7 and −9 in Non-Small Cell Lung Cancer and Controls

Marker^a^	Median	Range	*P* ^b^	AUC (95% CI)
MMP-1 (pg/mL)				
Control	6209.72	935.32–23,960.37	0.364	0.538 (0.457–0.620)
Healthy	4730.11	1081.81–23,635.33	0.045	
Benign	7483.33	935.32–23,960.37	0.974	
NSCLC	6653.85	1450.34–41,668.33		
NSCLC I + II	6046.96	1784.41–30,180.33	0.772	
NSCLC III + IV	6972.73	1450.34–41,668.33	0.324	
MMP-7 (pg/mL)				
Control	22,130.10	5026.14–79,977.27	0.014	0.604 (0.523–0.685)
Healthy	21,010.07	8893.61–48,743.79	0.004	
Benign	22,739.58	5026.14–79,977.27	0.109	
NSCLC	26,485.45	5383.18–79,809.13		
NSCLC I + II	26,165.87	13,727.30–54,573.51	0.465	
NSCLC III + IV	26,680.26	5383.18–79,809.13	0.007	
MMP-9 (ng/mL)				
Control	194.84	11.07–3611.59	<0.001	0.739 (0.670–0.809)
Healthy	177.86	52.79–800.30	<0.001	
Benign	200.28	11.07–3611.59	<0.001	
NSCLC	348.04	21.06–1941.08		
NSCLC I + II	346.03	116.92–1941.08	0.002	
NSCLC III + IV	364.62	21.06–1914.00	<0.001	

*NSCLC* Non-Small Cell Lung Cancer

^a^Sample size: Control *N* = 110 (Healthy *N* = 35, Benign *N* = 75), NSCLC *N* = 83 (NSCLC I + II *N* = 24, NSCLC III + IV *N* = 59)

^b^Mann-Whitney U test for the comparison between the cancer and control groups, for subtypes of controls versus NSCLC, and NSCLC stages versus all controls

In terms of AUC, MMP-9 was the marker that better discriminated NSCLC from all control individuals (AUC 0.739), while MMP-1 and MMP-7 demonstrated poor diagnostic capability (AUC of 0.538 and 0.604, respectively).

### Analysis of serum MMP-1, MMP-7 and MMP-9 regarding NSCLC stage

Marker levels were also evaluated according to cancer stage (Table 4). A trend towards increased marker levels was observed with cancer progression, particularly for MMP-1 and MMP-9. Consequently, all three MMPs exhibited significant differences between late-stage NSCLC and all controls. MMP-9 was the only molecule that could discriminate NSCLC at early stages from control individuals (*P* = 0.002), suggesting its usefulness in early NSCLC diagnosis.

### Multi-MMP panel for NSCLC classification

Prior to evaluate the usefulness of the combination of MMPs for NSCLC discrimination, univariate logistic regression models of MMPs and demographic variables was accomplished to assess their predictive value for NSCLC (Additional file 2). Univariate regression demonstrated a significant association between being male, older aged, current or past smoking history, and high levels of MMP members MMP-7 and MMP-9. As expected, the strongest relation with lung malignancy was observed for MMP-9, with an unadjusted OR of 8.86 (95% CI 3.17–24.76). When the predictive variables were included in multivariate regression models, MMP-9 remained the unique MMP significant in NSCLC prediction, as well as older age and having a positive smoking history.

Since MMP-9 resulted the marker that better discriminated cancer from controls, this molecule was combined with MMP-1 and/or -7 with the intention of increasing its diagnostic performance. Logistic regression was applied to the 177 patients with data on MMP-1, MMP-7 and MMP-9, and information on gender, age and smoking variables, to construct multimarker classification algorithms including gender, age and smoking history as confounders. Discriminatory capability by means of AUC was estimated for MMP-9 and each combination, as well as the diagnostic parameters based on the Youden index (Table 5).

**Table 5:** Comparison of Diagnostic Performance of MMP-9 and its Combination with MMP-1 and/or MMP-7 for Non-Small Cell Lung Cancer Detection

Model^a^	AUC	Sensitivity % (95% CI)^b^	Specificity % (95% CI) ^b^	Youden Index
MMP-9 + Gn + Ag + Smk	0.787 (0.720–0.845)	85.54 (76.1–92.3)	64.89 (54.4–74.5)	>0.419
MMP-9 + MMP-1 + Gn + Ag + Smk	0.787 (0.720–0.845)	85.54 (76.1–92.3)	64.89 (54.4–74.5)	>0.421
MMP-9 + MMP-7 + Gn + Ag + Smk	0.786 (0.718–0.844)	81.93 (72–89.5)	64.52 (53.9–74.2)	>0.413
MMP-9 + MMP-1 + MMP-7 + Gn + Ag + Smk	0.788 (0.720–0.845)	81.93 (72–89.5)	64.52 (53.9–74.2)	>0.415

*Gn* Gender, *Ag* Age, *Smk* Smoking history

^a^Logistic regression models were elaborated including the 177 patients with data on MMP-1, MMP-7 and MMP-9, and information on gender, age and smoking variables

^b^Sensitivity and specificity calculated based on Youden Index

The AUC for MMP-9 adjusted by gender, age and smoking history (0.787) practically did not increase when combined with either MMP-1, MMP-7 or both MMP-1 and MMP-7 (AUC of 0.787,0.786 and 0.788, respectively), indicating that these MMPs do not complement MMP-9 to separate cancer patients from controls. In relation to the diagnostic parameters, MMP-9 combination with demographic characteristics rendered notable sensitivity and specificity (85.54 and 64.89%, respectively), identical to those obtained when MMP-1 is included in the classification model. On the other hand, the inclusion of MMP-7 or MMP-1 and MMP-7 diminished sensitivity (81.93%) and specificity (64.52%) in both cases.

## Discussion

Initially MMPs were considered only relevant to invasion and metastasis due to their relation with the cleavage of structural components of the ECM. However, through their action on other non-ECM substrates, including growth factors and receptors, cytokines, chemokines and cell-adhesion molecules, they regulate important cancer processes such as cell growth, apoptosis, tumor angiogenesis and evasion of immune surveillance [5, 18].

On the basis of their lung-tumorigenesis promoting functions [10–14], the MMP members MMP-1, MMP-2, MMP-7, MMP-9 and MMP-10 may result promising biomarkers for lung cancer. Furthermore, these MMPs are commonly reported as over-expressed in lung tumor tissues as compared to corresponding normal tissues in NSCLC patients [19–22], however, little is known about their diagnostic value. The goal of this study was to evaluate serum MMP-1, -2, -7, -9, -10 and TIMP-1 in discriminating NSCLC from non-cancerous controls.

Two of the five MMPs first assayed in the initial sample population were discarded based on poor AUC values (MMP-2: 0.446; MMP-10: 0.564), showing no value for NSCLC discrimination. The work of Kanoh et al. [23] compared serum MMP-2 levels in NSCLC patients and healthy controls, finding no significant alteration in MMP-2 concentration. In relation to serum MMP-10, its potential as a tumor marker for NSCLC diagnosis has not been earlier addressed, to our knowledge. Available literature reports higher expression in lung tumor tissues [22], although reduced MMP-10 mRNA levels have been reported too [24].

MMP-1, MMP-7 and MMP-9 in the initial study set described relatively elevated serum levels in NSCLC patients versus controls, and that prompted us to determine their diagnostic value in a larger population comprising benign pathologies of the lung that must also be discerned from NSCLC patients. Overall, MMP-1 levels in NSCLC were higher than that of the control group, although discrimination was poor as evidenced by the AUC value of 0.538. MMP-1 studies on its diagnostic usefulness for NSCLC are scarce. The study of Li et al. [25] in plasma of lung cancer patients confirmed higher MMP-1 levels in NSCLC regarding healthy individuals, but diagnostic capability seemed limited too. Clearly, MMP-1 concentration is enhanced in the benign group, abrogating the discrimination from NSCLC, in agreement with the observation of Rosas et al. [26], who propose MMP-1 as a blood marker for Idiophatic Pulmonary Fibrosis (IPF). Regarding tumor stage, higher levels were found in advanced stages versus controls, although this increase did not reach statistical difference. Li et al. [25] also observed considerable higher levels for stages III and IV, finding an association of both elevated plasma levels and tissue up-regulation with poor survival rates. These results suggest that MMP-1 could be implicated in the late stages of the tumor process.

Determination of MMP-7 levels in NSCLC, benign and healthy controls displayed a statistical significant increase in cancer patients, though a moderate discriminatory capacity was found for MMP-7 (AUC: 0.604). As for MMP-1, studies on MMP-7 point to a role of this MMP as marker of IPF rather than NSCLC diagnosis [26, 27]. Ulivi et al. [27] analyzed the diagnostic potential of MMP-7 in discriminating between NSCLC and other lung diseases (chronic obstructive pulmonary disease, asthma,...), healthy donors and non-specific interstitial pneumonias. In spite of significantly higher levels in NSCLC than in lung diseases and donors, similar levels to that of interstitial pneumonias derived in an AUC of 0.64.

MMP-9 is one of the most studied MMPs in lung cancer, with several reports addressing its potential as diagnostic marker. In our study, significantly elevated serum MMP-9 was observed in patients with malignancy regarding both healthy controls and patients with benign pathologies, offering the best diagnostic capacity for NSCLC with an AUC value of 0.739. These findings are in line with other studies reporting significantly higher MMP-9 levels in serum of NSCLC patients compared with non-malignant lung diseases or healthy controls [28–30]. Especially relevant diagnostic terms are presented in the study by Zhang et al. [28], who reported an AUC of 0.882 in differentiating NSCLC from healthy and non-malignant lung patients, and by Fiorelli et al. [29], who proposed the combined use of MMP-9 with PET to diagnose malignancy in indeterminate pulmonary lung lesions. This study described for PET-negative patients a sensitivity of 94% and specificity of 100% in classifying these lesions.

Regarding tumor stage we found a not significant increase in MMP-9 levels with advanced stages. These results are consistent with the described trend of highest levels in metastatic disease [29, 30]. The association between serum MMP-9 and gender was also previously reported [28], with males presenting higher levels.

The balance between MMPs production and their regulators is crucial to avoid deregulated ECM turnover or cell growth, which would result in disease [16, 18]. In this work we also aimed to consider the effect of TIMP-1 on MMP-9 levels by defining the ratio MMP-9/TIMP-1 in the initial study set. We found that TIMP-1 levels were moderately elevated in NSCLC patient’s sera, as previously observed by Jumper et al. [30], although these authors found a significant increase. Nevertheless, the MMP-9/TIMP-1 ratio did not improve the metalloprotease discrimination of NSCLC, with a loss in AUC from 0.852 to 0.781, therefore it was not further evaluated. The relationship between MMP-9 and its inhibitor remains undetermined, as both no correlation [30] and a significant correlation [31] have been reported in serum of lung cancer patients, and could be in part attributable to other non-MMP inhibiting activities of TIMP-1.

Among all the MMPs analyzed in our study, including the MMP-9/TIMP-1 ratio, MMP-9 displayed the best diagnostic accuracy for NSCLC. Disappointingly, this discrimination seems not good enough for clinical application. Numerous reports in the literature have evaluated the utility of combined tumor-related markers for lung cancer detection [32–34] and, consequently, we tested whether MMP-1, MMP-7 and MMP-9 could provide complementary discrimination and improve the performance of MMP-9. Logistic regression was used to develop panels composed of 2 or 3 markers, in conjunction with the variables gender and age, to distinguish between NSCLC cases and controls. Two-marker panels based on MMP-9 with either MMP-1 or MMP-7 offered identical AUC-based discrimination, but did not improve that of MMP-9 adjusted by confounders.

Several publications have recently examined the additional value of serum MMPs to protein markers on NSCLC detection models, as Farlow et al. [35] and Bigbee et al. [36]. The first included MMP-2 as part of a multi-analyte test for prediction of NSCLC diagnosis, reporting significantly higher concentrations in the control group compared to NSCLC, with an AUC value of 0.705. Inclusion of rheumatology inflammatory conditions and resected nodules as non-cancerous controls could account for the discrepancy with our results. That study also evaluated MMP-9 and TIMP-1 among the candidate markers, though these were not selected as statistically relevant by the algorithm employed. Another multiplexed-marker panel for complementing CT in discriminating NSCLC from cancer-free controls was proposed by Bigbee et al. [36]. Among the 70 cancer-associated proteins evaluated, MMP members MMP-1, -7 and -9 were considered, although they were not included in the final diagnostic rule. On the contrary, Izbicka et al. [37] in plasma from NSCLC and healthy controls proposed a 5-marker panel whose diagnostic performance reached 95% sensitivity and 79% specificity, resulting MMP-9 one of the relevant proteins in the discriminative model.

## Conclusions

Our study on the relevance of MMPs in NSCLC diagnosis indicates that MMP-2 and MMP-10 exhibit a poor discrimination of NSCLC versus healthy controls, while MMP-1 and -7 differentiate NSCLC from healthy controls, but not from benign diseases. On the other hand, a notable discriminatory performance was reached for MMP-9 when determined jointly with demographic variables. Based on this evidence, MMP-9 would be a suitable candidate to be included in other models or assays to improve the accuracy in NSCLC diagnosis.

## Additional files

Additional file 1: Patient Demographics and Classification of Non-Small Cell Lung Cancer in Initial Study Set. (DOC 43 kb)

Additional file 2: Univariate and multivariate logistic regression analysis of MMP-1, MMP-7, MMP-9, gender, age and smoking history regarding NSCLC. (DOCX 13 kb)

## Abbreviations

ADC: Adenocarcinoma
AUC: Area under the ROC curve
ECM: Extracellular matrix
IPF: Idiopathic pulmonary fibrosis
LCC: Large cell carcinoma
MMPs: Matrix metalloproteases
NSCLC: Non-small cell lung cancer
ROC: Receiver operating characteristic
SCC: Squamous cell carcinoma
TIMP: Tissue inhibitors of metalloproteinases
