# Supplementary material for: Relevance of matrix metalloproteases in non-small cell lung cancer diagnosis
Source: BMC Cancer. 2017 Dec 5;17:823. doi: 10.1186/s12885-017-3842-z (PMC5718060; doi:10.1186/s12885-017-3842-z)
Supplement: Supplementary file 1 — Patient Demographics and Classification of Non-Small Cell Lung Cancer in Initial Study Set. (DOC 43 kb) [file 12885_2017_3842_MOESM1_ESM.doc]

| **Additional File 1:** Patient Demographics and Classification of Non-Small Cell Lung Cancer in Initial Study Set | | | |
| --- | --- | --- | --- |
|  | | **Cases (n=19)** | **Healthy (n=19)** |
| **Gender** | |  |  |
| Male | | 15 (78.9%) | 12 (63.2%) |
| Female | | 4 (21.1%) | 7 (36.8%) |
| **Age** | |  |  |
| Median | | 58 | 58 |
| Range | | 37-86 | 40-82 |
| **Smoking status** | |  |  |
| Yes | | 16 (84.2%) | 13 (68.4%) |
| No | | 3 (15.8%) | 6 (31.6%) |
| **Diagnosis** | |  |  |
| Healthy | |  | 19 |
| NSCLC Stage | | 19 |  |
|  | I | 5 (26.3%) |  |
|  | III | 5 (26.3%) |  |
|  | IV | 9 (47.4%) |  |
| Abbreviations: NSCLC=Non-Small Cell Lung Cancer | | | |
