# Supplementary material for: Relevance of matrix metalloproteases in non-small cell lung cancer diagnosis
Source: BMC Cancer. 2017 Dec 5;17:823. doi: 10.1186/s12885-017-3842-z (PMC5718060; doi:10.1186/s12885-017-3842-z)
Supplement: Supplementary file 2 — Univariate and multivariate logistic regression analysis of MMP-1, MMP-7, MMP-9, gender, age and smoking history regarding NSCLC. (DOCX 13 kb) [file 12885_2017_3842_MOESM2_ESM.docx]

| **Additional File 2: Univariate and multivariate logistic regression analysis of MMP-1, MMP-7, MMP-9, gender, age and smoking history regarding NSCLC.** | | | | | | |
| --- | --- | --- | --- | --- | --- | --- |
| **Variables** | **Gender (Male) ^a^** | **Age ^a^** | **Smoking (Yes) ^a^** | **logMMP-1 ^a^** | **logMMP-7 ^a^** | **logMMP-9 ^a^** |
| **Gender (men)** | 2,814 (1,402-5,648)  0,004 |  |  |  |  |  |
| **Age** **(years)** |  | 1,038 (1,013-1,063)  0,003 |  |  |  |  |
| **Smoking history (yes)** |  |  | 4,244 (1,882-9,567)  <0,001 |  |  |  |
| **MMP-1** |  |  |  | 1,770 (0,691-4,535)  0,234 |  |  |
| **MMP-7** |  |  |  |  | 6,432 (1,296-31,926)  0,023 |  |
| **MMP-9** |  |  |  |  |  | 8,860 (3,170-24,762)  <0,001 |
| **MMP-9 + Gn + Ag + Smk** | 1,283 (0,517-3,185)  0,591 | 1,046 (1,018-1,074)  0,001 | 4,611 (1,590-13,371)  0,005 |  |  | 8,115 (2,789-23,612)  <0,001 |
| **MMP-9 + MMP-1 + Gn + Ag + Smk** | 1,284 (0,517-3,189)  0,590 | 1,046 (1,018-1,075)  0,001 | 4,614 (1,590-13,386)  0,005 | 0,965 (0,325-2,864)  0,949 |  | 8,176 (2,739-24,409)  <0,001 |
| **MMP-9 + MMP-7 + Gn + Ag + Smk** | 1,287 (0,519-3,195)  0,586 | 1,040 (1,010-1,070)  0,008 | 4,113 (1,413-11,971)  0,009 |  | 2,962 (0,452-19,407)  0,258 | 8,424 (2,880-24,640)  <0,001 |
| **MMP-9 + MMP-1 + MMP-7 + Gn + Ag + Smk** | 0,773 (0,312-1,919)  0,579 | 1,040 (1,010-1,070)  0,008 | 4,104 (1,409-11,953)  0,010 | 0,877 (0,290-2,654)  0,816 | 3,073 (0,455-20,758)  0,249 | 8,669 (2,874-26,148)  <0,001 |
| Abbreviations: Gn=Gender, Ag=Age, Smk=Smoking history | | | | | | |

^a^ OR (95% CI) and *P-value* are provided for each variable. Logistic regression models were elaborated including the 177 patients with data on MMP-1, MMP-7 and MMP-9, and information on gender, age and smoking variables
